# Er:YAG Laser and Fractured Incisor Restorations: An *In Vitro* Study

**DOI:** 10.1155/2012/617264

**Published:** 2012-10-03

**Authors:** C. Fornaini, S. Petruzzella, R. Podda, E. Merigo, S. Nammour, P. Vescovi

**Affiliations:** ^1^Oral Medicine and Laser-Assisted Surgery Unit, Dental School, Faculty of Medicine, University of Parma, Via Gramsci 14, 43126 Parma, Italy; ^2^Laser Unit, Dental Sciences Department, Faculty of Medicine, University of Liège, Quai Godefroid Kurth, 45, 4020 Liège, Belgium

## Abstract

*Introduction*. The aim of this study was to analyse the effects of an Er:YAG laser on enamel and dentine in cases of dental restorations involving fractured teeth, utilizing the dental fragment. *Materials and Methods*. Seventy-two freshly extracted bovine incisors were fractured at the coronal level by using a hammer applied with a standardized method, and the fragment was reattached by using three different methods: Er:YAG laser, orthophosphoric acid, and laser plus acid. The different groups were evaluated by a test realized with the dynamometer to know the force required to successfully detach the reattached fragment and by a microinfiltration test by using a 0.5% methylene blue solution followed by the optic microscope observation. *Results*. The compression test showed only a slight difference between the three groups, without any statistical significance. The infiltration test used to evaluate the marginal seal between the fracture fragment and the tooth demonstrated that etching with Er:YAG laser alone or in combination with orthophosphoric acid gives better results than orthophosphoric acid alone, with a highly significant statistical result. *Discussion*. Reattaching a tooth fragment represents a clinically proven methodology, in terms of achieving resistance to detachment, and the aim of this work was to demonstrate the advantages of Er:YAG laser on this procedure. *Conclusion*. This “*in vitro*” study confirms that Er:YAG laser can be employed in dental traumatology to restore frontal teeth after coronal fracture.

## 1. Introduction

Dental traumatology is a multidisciplinary branch of dentistry that requires a number of specific skills where, in cases of emergency, decisions have to be made within a limited timeframe and with effects that are only possible to be evaluated at a later date [1]. The technique of tooth fragment reattachment should be adapted, both in cases of simple coronal fracture (enamel and superficial dentine) as well as in complicated coronal fracture (deep dentine with pulp exposure) [2].

While in the first case the fragment may even be reattached immediately, in complicated coronal fractures the main concern should be the protection of the pulp and not necessarily the fragment, which should be kept hydrated in the fridge in a container marked with the patient's full name and the date of the trauma. The solution in the container should be changed at regular intervals and the seal checked, since in some cases, fragments may be stored for some months before being reattached.

The field of adhesive dentistry was born in 1955 by Buonocore with the description of the utilisation of orthophosphoric acid and composite resin in order to obtain restorations with high bond strength and reduced microleakage [3, 4]. In 1990 laser technology was introduced in conservative dentistry by Hibst and Keller, who described the possibility to use an Er:YAG laser as an alternative to conventional instruments such as the turbine and micromotor [5, 6]. Widespread interest in employing this new technology is related to its significant number of advantages, as described in several scientific studies. In fact, Er:YAG laser technology allows for efficient ablation of hard dental tissues, thanks to the affinity of its wavelength to water and hydroxyapatite, without the risk of micro- and macro-fractures which have been observed by using conventional rotating instruments [7–9]. The dentin surface treated by laser appears clean, without a smear layer and with the tubules open and clear [10].

Thermal elevation in the pulp, recorded during Er:YAG laser irradiation, is less to that recorded by using turbine and micromotor in the same conditions of air/water spray [11, 12]. This wavelength has an antimicrobial decontamination effect on the treated tissues, which destroys both aerobic and anaerobic bacteria [13]. The most interesting aspects of this new technology are related to the goals of the modern conservative dentistry: “minimally invasive dentistry” and “adhesive dentistry.” Er:YAG lasers can reach spot dimensions smaller than 1 mm, which enables the possibility to make a selective ablation of the affected dentin while preserving the sound tissue in order to realize very limited restorations [14].

Several in vitro studies demonstrated that the preparation of enamel and dentine by Er:YAG laser followed by orthophosphoric acid etching enhances the effectiveness in terms of reduced microleakage and increased bond strength [15]. Several authors have proposed the utilisation of laser technology also for the restoration of the frontal teeth fractured by traumatic events [16].

The aims of this in vitro study were to test the usefulness of the Er:YAG laser in the treatment of tooth fractures, by evaluating strength and microleakage of restorations obtained by bonding the broken fragment directly to the tooth.

## 2. Materials and Methods

**Figure 1:**
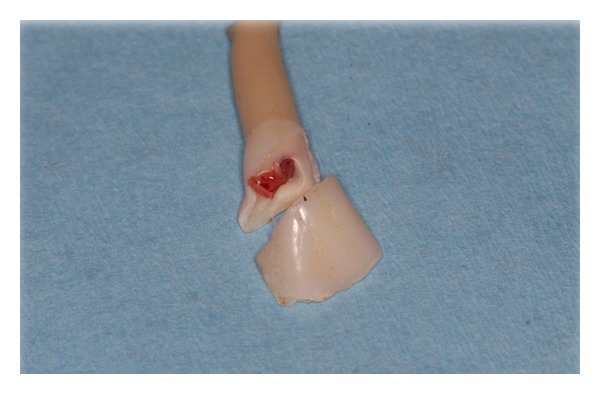
Fracture produced in the bovine tooth by hammer.

Ninety-six bovine incisors were extracted by removal of the periodontal ligament with a sharp blade and subsequently carefully cleansed with sodium hypochlorite in 2% distilled water solution (Amukine 20 mL/1000 mL of distilled water). Samples were stored in a fridge at 4°C in a physiological solution, changed once per week. The teeth, removed from the container with anatomical pincers, were individually positioned in a tool table vice clamped at belt level with two felt pads. A 100 g weight hammer was employed to fracture the dental crowns, using one or two clean blows (Figure 1).

Each fractured tooth was replaced with its own fragment immersed in a container filled with 0.9% physiological solution. Fracture procedure produced more than one fragment in twenty-four teeth, resulting in exclusion from the study. The remaining seventy-two teeth (consisting of one single fracture fragment) were randomly subdivided into two groups of thirty-six teeth each (group 1 and group 2). Each group was subdivided into subgroups A-B-C, consisting of twelve dental elements.

### 2.1. Groups 1A and 2A (Laser Etching)

- Laser etching was realized with an Er:YAG laser (Fidelis Plus III, Fotona, Slovenia) with the following parameters: 150 mJ, 10 Hz, 1.5 W, VSP (100 *μ*sec) pulse duration, 29.9 J/cm^2^ fluence, with water/air spray and an R02 handpiece (angle of 90° with an 0.80 mm spot at a distance of 1.2 mm). The procedure was made on both the fracture fragment and the tooth. In order to check the energy output from the handpiece, even though the articulated arm delivering system has a negligible loss of energy, a power meter (Ophir Nova II, thermal head F150A, Israel) was used.
- The fragments and teeth were dried with air spray for 15 s and then treated with applying adhesive (Prime & Bond NT Dual Cure, DENTSPLY Caulk, Milford, CT, USA) in a single step with a single-use brush, both on the tooth and the fragment.
- After leaving the tooth and fragment for 15 s, they were both photopolymerized by lamp (3 M DENTSPLY De Trey, Konstanz, Germany) for 20 s.
- A thin layer of Estelite Flow Quick composite (Tokuyama Dental Corp., Japan) was applied to the tooth.
- The fragment was positioned to the tooth by hand and maintained in this position during polymerization with the halogen lamp (3 M DENTSPLY) for 30 s on the vestibular face and 30 s on the palatal face.
- The restored teeth were replaced in physiological solution inside proper containers.

### 2.2. Groups 1B and 2B (Acid Etching)

- The fractured surfaces of twenty-four teeth with their own fracture fragments were treated by the application of orthophosphoric acid gel at 37% concentration for 30 s, (both tooth and fragment) and then rinsed with water spray for 15 s.

The steps (b), (c), (d), (e), and (f) were the same as described for the groups 1A and 2A.

### 2.3. Groups 1C and 2C (Acid and Laser Etching)

- The fractured surfaces of twenty-four teeth with their respective fracture fragments were firstly treated with an Er:YAG laser (Fotona) with the parameters previously described. Fragments and teeth were dried with an air spray for 15 s and etched with orthophosphoric acid gel at a concentration of 37% for 30 s (both tooth and fragment).

The steps (b), (c), (d), (e), and (f) were the same as described for the groups 1A, 2A, 1B, and 2B.

**Compression Test**

The teeth of group 1 were used to measure the force required to successfully detach the reattached fragment, using a dynamometer (PCE series SH 500, PCE Group, Lucca, Italy) with a resolution of 0.1 N, with ±0.5% accuracy, mounted on a SLJ50 manual stand made by the same company.

The procedure was performed as follows.

**Figure 2:**
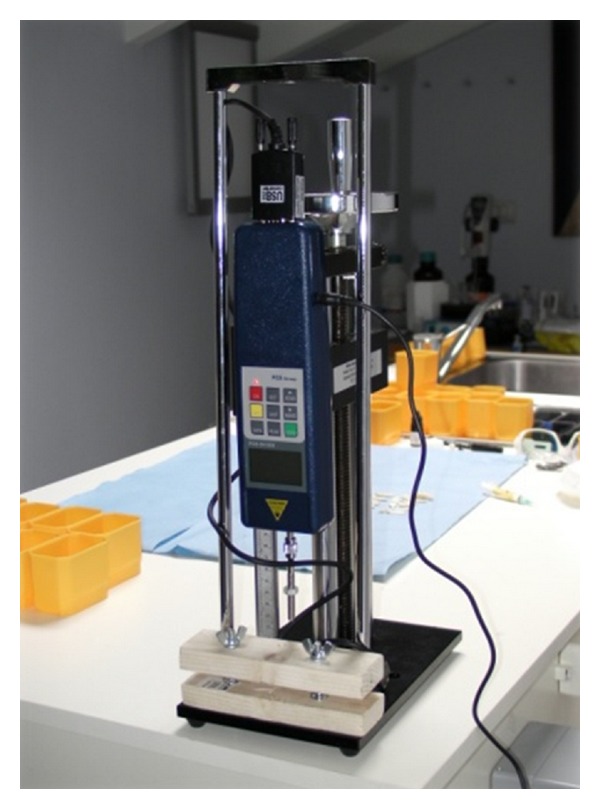
Dynamometer used to make the compression tests.

- Each tooth was clamped, at neck level, in a wooden vice mounted at the base of the stand.
- The dynamometer was brought into contact with the crown and a gradually increasing compressive force was applied until the detachment of the tooth fragment (Figure 2).
- The procedure was recorded on a chart using appropriate software applied to the above-mentioned dynamometer (software for PCE SH500, PCE Group, Lucca, Italy).

**Microleakage**

The teeth of group 2 were used to analyse the microleakage as follows.

**Figure 3:**
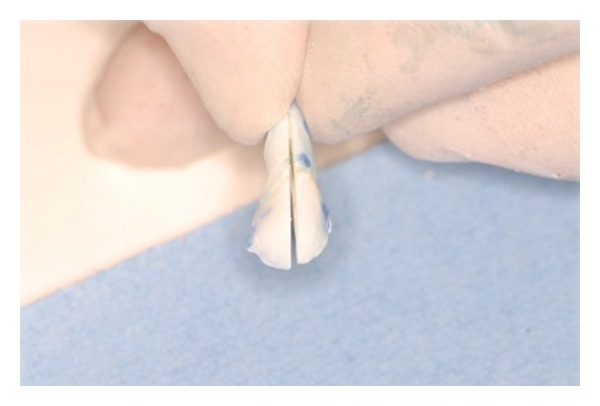
Tooth cut in 2 parts in the vestibule-lingual direction in order to obtain 2 symmetrical fragments.

1. The radicular apex was hermetically sealed with wax.
2. Each tooth was waterproofed up to 0.5 mm from the edge of the fracture, through the application of two coats of transparent nail varnish (nitrocellulose dimethyl acetone MPH air, Rimini, Italy) applied with a drying interval of 10 min.
3. Each sample was immersed in a 0.5% methylene blue solution for 12 h.
4. Every sample was rinsed with tap water and then replaced in a container with physiological solution.
5. After about 1 h, the teeth were removed, dried firstly with absorbent paper and then with an air spray.
6. The previously applied transparent varnish was removed with acetone, and any remaining trace of varnish was eliminated with a rubber tip.
7. The root was eliminated by using a diamond disk approximately 4 mm from the amelocemental junction, and the remaining part of the tooth was cut in 2 parts in the vestibule-lingual direction in order to obtain 2 symmetrical fragments (Figure 3).
8. Each fragment was examined with an optical microscope (Novex zoom Stereo RZ, Euromex Microscopen, the Netherlands) in order to evaluate the penetration of methylene blue using a scale as described in the ISO technique. Two different operators blindly conducted the examination, and the criteria of the scores are described in Table 1.

## 3. Statistical Analysis 

Statistical analysis was performed with the Chi-squared test, normally used to compare the tallies or counts of categorical responses between two (or more) independent groups, and a one-way analysis of variance (ANOVA) test, used in cases where there are more than two groups; statistical significance was achieved for *P* > 0.05. 

## 4. Results

Forces (N) required for the detachment of teeth fragments are shown in Table 2.

Statistical analysis of the fracture forces under compression for each subgroup of group 1 (1A-1B-1C) did not reveal any statistically significant differences (*P* = 0.7227) (Table 3). The results of the infiltration are showed in the Table 4.

The comparison of the three different etching methods, considering microleakage in terms of low degree (degrees 0-1) and high degree (degrees 2-3) of infiltration showed a highly significant result (*P* < 0.0001) with 0 high-degree infiltration samples for the laser etching (group 1A), 14 high-degree infiltration samples for group 1B, and 2 high-degree infiltration samples for group 1C.

## 5. Discussion 

Reattaching a tooth fragment represents a clinically proven methodology, in terms of achieving resistance to detachment; the aim of this work was to measure, in quantitative terms, the adherence of the fracture fragment to the tooth as well as the seal in the interface zone by an infiltration test. In the literature, there is no similar protocol describing the use of laser etching as applied to tooth fragment reattachment techniques. The infiltration test used demonstrated that etching with an Er:YAG laser alone or with orthophosphoric acid gave better results than by etching with orthophosphoric acid alone, in a statistically significant manner. All samples demonstrated a degree of marginal infiltration even if

1. in the samples etched with the Er:YAG laser alone, the majority of the samples do not show evidence of dye infiltration (23/24 observed with optical microscope);
2. at the level of the adhesion area of the samples etched with the Er:YAG laser and orthophosphoric acid, an absence of infiltration was noted in 21 out of 24 samples;
3. in samples etched with orthophosphoric acid alone, only 10 out of 24 samples showed no marginal infiltration, while 14 out of 24 samples showed evidence of infiltration which reached the pulp chamber (degree 3).

Results of the infiltration test showed that the use of etching with Er:YAG laser gave a highly significant result compared to the use of orthophosphoric acid, and furthermore, that the use of the Er:YAG laser alone compared to Er:YAG laser and orthophosphoric acid demonstrated evidence of less infiltration, even if not by a statistically significant extent.

It was decided to use, in order to assess the adherence comparing the sample groups, a compression test instead of the flexural test normally utilised in these types of studies. The reason is that this “in vitro” situation is more similar to the mechanical forces applied to incisors during “in vivo” mastication.

The results showed only a slight difference between the three differently etched subgroups in group 1. Group B, etched with acid only, had the best values, as reported in Table 4, for maximum (1286 N), minimum (336 N), and average (611 N) while Group C (laser + acid) had the worst values for maximum (1115 N), minimum (166 N), and average (551 N).

The differences between all the groups were not statistically significant (*P* < 0.1) and this means that the use of an Er:YAG laser combined with acid etching gives the same bond strength as the acid-only etched teeth.

The result of this work also holds significance in regard to the controversial role of orthophosphoric acid etching when using lasers for cavity preparation. Many authors support the necessity of orthophosphoric acid etching also after Er:YAG irradiation [17] while others have demonstrated the efficacy of laser preparation alone in terms of adhesion [18]. 

In fact, there is no evidence of any significant difference for conditioning enamel and dentine between using the Er:YAG laser alone or in combination with orthophosphoric acid and, according to these results, Er:YAG laser should be the first choice for conditioning enamel and dentine.

## 6. Conclusion

The Er:YAG laser may be used in conservative dentistry as an alternative to conventional instruments and in association with orthophosphoric acid, with several advantages, such better strength bond [19], reduced microleakage [20], and also lower discomfort and higher patient satisfaction [21].

This “in vitro” study, even if considered as preliminary due to the limited number of samples, confirms that it can be employed also in dental traumatology, to restore frontal teeth after coronal fracture, with the advantage of improved adhesion of the dental fragment to the tooth, in particular by decreasing microleakage.

In fact, all the microinfiltration tests made on bovine-extracted samples demonstrated a statistically significant difference between the laser-treated and non-laser-treated groups. The compression test did not show significant differences between the sample groups, indicating that Er:YAG laser does not reduce the adhesion of composite resin when compared to the traditional instruments.

Regarding the methodology of this study, it would be interesting also to analyse samples with SEM in order to see, both in the teeth and fragments, the ultrastructural differences by using different preparation and etching techniques.

## Figures and Tables

**Table 1:** Criteria used by the two blind operators to assign the scores for infiltration evaluation.

Extent of dye recorded	Code
Absence of penetration	0
Limited penetration on the enamel portion of the wall	1
Penetration which also involves the dentine portion of the walls without affecting the roof of the pulp chamber	2
Penetration which reaches as far as the roof of the pulp chamber and affects it	3

**Table 2:** Forces recorded by the traction test to obtain the detachment of the fragments.

Sample	Group A (laser)	Group B (acid)	Group C (laser + acid)
1	500	1266	292
2	546	436	336
3	626	336	796
4	614	860	836
5	456	932	166
6	586	716	607
7	1036	606	315
8	460	616	1115
9	322	422	490
10	620	646	779
11	484	436	323
12	638	486	736

**Table 3:** Statistical analysis of the forces in the traction test.

	Group A (laser)	Group B (acid)	Group C (laser + acid)
Mean	598,33	648,166	565,583
Standard deviation (SD)	182,62	270,46	288,19
Sample size (*N*)	12	12	12
Std. Error of mean (SEM)	52,717	78,074	83,193
Lower 95% conf. limit	482,80	476,33	382,48
Upper 95% conf. limit	714,86	820,01	748,69
Minimum	322,00	336,00	166,00
Median (50th percentile)	600,00	611,00	551,50
Maximum	1036,00	1286,00	1115,00
Normality test KS	0,2527	0,1699	0,2038
Normality test *P* value	0,0331	>0,10	>0,10
Passed normality test?	No	Yes	Yes

**Table 4:** Results obtained by the infiltration test.

Sample	Group A (laser)		Group B (acid)		Group C (laser + acid)	
	Vestibular	Palatal	Vestibular	Palatal	Vestibular	Palatal
1	0	0	0	0	0	0
2	0	0	0	0	0	0
3	0	0	3	3	0	0
4	0	0	3	3	0	0
5	0	0	3	3	0	0
6	0	0	0	0	0	0
7	0	0	3	3	0	0
8	0	0	3	3	0	1
9	0	0	0	0	0	0
10	0	0	0	0	3	3
11	0	1	3	3	0	0
12	0	0	3	3	0	0
